# Safety and Immunogenicity of Single-Dose of Adsorbed Tetanus Vaccine in Adults Aged 18–44 Years: Randomized, Double-Blind, Positive-Controlled Phase I/III Clinical Trial

**DOI:** 10.3390/vaccines13090972

**Published:** 2025-09-13

**Authors:** Zhiqiang Xie, Liyong Yuan, Yaping Qiao, Wangyang You, Yurong Li, Taotao Zhu, Wei Zhang, Lili Huang, Jiebing Tan, Xiaocan Jia, Zhe Li, Feng Xue, Xiaojuan Lian, Yanxia Wang

**Affiliations:** 1 Henan Provincial Center for Disease Control and Prevention, Zhengzhou 450016, China; xiezqshang@163.com (Z.X.); dsrt12345@163.com (W.Y.); zwzzu@163.com (W.Z.); ilovehongshupian@163.com (L.H.); tanjiebingz@163.com (J.T.); 2 National Institutes for Food and Drug Control, Beijing 102629, China; yuanly@nifdc.org.cn (L.Y.); lizhe@nifdc.org.cn (Z.L.); 3 Sinovac Holding Group Co., Ltd., Beijing 100085, China; qiaoyp6109@sinovac.com (Y.Q.); liyr8134@sinovac.com (Y.L.); zhutt@sinovac.com (T.Z.); 4 Department of Epidemiology and Biostatistics, College of Public Health, Zhengzhou University, Zhengzhou 450001, China; jxc@zzu.edu.cn; 5 Sinovac Life Sciences Co., Ltd., Beijing 102601, China; xuefeng@sinovac.com

**Keywords:** tetanus, vaccine, immunogenicity, safety, clinical trial

## Abstract

**Background**: The persistence of non-neonatal tetanus (non-NT) highlights the necessity of adult booster vaccines and post-traumatic prophylaxis. This Phase I/III clinical trial aimed to evaluate the safety and immunogenicity of a new adsorbed tetanus vaccine. **Methods**: A randomized, double-blind, positive-controlled clinical trial was conducted in Henan Province, China. A total of 1258 healthy participants aged 18–44 years (60 in Phase I and 1198 in Phase III) were enrolled, with no history of tetanus infection, or tetanus toxoid-containing vaccines (TTCVs) vaccination within the past 10 years. The participants were randomly assigned at a 1:1 ratio to receive a single dose of either the investigational vaccine or the licensed control vaccine. The Phase III clinical trial was initiated subsequent to the 7-day safety observation period following vaccination in the Phase I trial. The objective of the Phase III clinical trial was to assess the non-inferiority of the seroconversion rate of tetanus antibodies at 30 days post-vaccination with the investigational vaccine compared to the control vaccine. Serum samples were collected prior to and at 30 days post-vaccination. Adverse events were monitored for 30 days, with serious adverse events (SAEs) followed up for 6 months post-vaccination. **Results**: The investigational group achieved a seroconversion rate of 99.48%, which was non-inferior to that of the control group (99.66%), with a negligible rate difference of −0.17% (95% confidence interval [CI]: −1.20%, 0.78%). The investigational group exhibited a significantly higher geometric mean concentration (GMC) of antibodies (4.721 IU/mL vs. 3.627 IU/mL, *p* < 0.0001). Among the susceptible participants, the seroconversion rates were 99.78% in the investigational group and 99.79% in the control group, respectively, with a non-inferior rate difference of −0.01% (95%CI: −1.06%, 0.97%). Furthermore, the investigational group showed a low incidence of adverse reactions (ARs) within 30 days post-vaccination (12.26%), which was comparable to that of the control group (13.65%). All the reported ARs were mild or moderate, and no SAEs were associated with the vaccination. **Conclusions**: The new adsorbed tetanus vaccine demonstrated favorable safety and comparable immunogenicity to the marketed control vaccine, with a significantly higher antibody GMC, supporting its clinical application in tetanus prevention.

## 1. Introduction

Tetanus, an acute and potentially fatal infection caused by *Clostridium tetani*, remains a persistent global public health burden. The bacterium enters through wound sites and produces tetanus toxin, a potent neurotoxin. Clinical manifestations typically occur within 3–21 days post-infection, with the majority of cases presenting within 14 days [1,2]. Affecting people across all age groups, the disease is characterized by generalized skeletal muscle rigidity and paroxysmal spasms, which can progress to life-threatening complications, such as laryngospasm, asphyxiation, pulmonary infection, and multiple organ failure [1,2]. Despite aggressive clinical management, tetanus is estimated to affect approximately 1 million individuals worldwide annually, resulting in 300,000–500,000 deaths [1]. Because humans lack natural immunity to tetanus, active immunization via vaccination is essential for inducing protective immunity against the tetanus toxin [2].

Despite the availability of effective vaccines, tetanus remains a substantial burden due to its high lethality, particularly in regions with inadequate immunization programs—including impoverished areas in low-income countries [3,4]. The World Health Organization (WHO) has recommended both sustaining the elimination of maternal and neonatal tetanus and expanding tetanus protection to all people [5]. Non-neonatal tetanus (non-NT) occurs predominantly as sporadic cases in rural and township areas. Due to the high rates of misdiagnosis and underdiagnosis, the reported incidence is likely to be significantly underestimated, indicating that non-NT remains a critical public health concern [1,6]. Currently, China lacks an epidemiological surveillance and reporting system for non-NT, resulting in an unclear national incidence profile [7,8].

Expanding vaccination coverage and ensuring vaccine efficacy are critical for mitigating the disease burden. Human tetanus toxoid-containing vaccines (TTCVs) include the absorbed tetanus toxoid vaccine, which serves as the cornerstone for post-traumatic prophylactic immunization, and combined vaccines, such as the adsorbed diphtheria and tetanus combined vaccine (DT) and the adsorbed diphtheria, tetanus, and acellular pertussis combined vaccine (DTaP) [1].

In many countries, adult vaccination programs are well-established, adhering to recommendations such as those issued by the US Centers for Disease Control and Prevention [9], the European Center for Disease Prevention and Control [10], and the Australian health authorities [11]. Most of these countries recommend a Td or Tdap booster every decade. As specified in *The Guideline for the Diagnosis and Treatment of Non-neonatal Tetanus (2024 Edition)*, the National Health Commission emphasizes the use of TTCVs over passive immunization for patients with unknown or incomplete vaccination histories [1]. Therefore, non-NT prevention in China will primarily rely on vaccines, supplemented by passive immunization agents, which is expected to drive an increase in the demand for TTCVs in the future.

A Phase I/III clinical trial was conducted to evaluate the immunogenicity and safety of a new tetanus vaccine manufactured by Sinovac Life Sciences Co., Ltd., Beijing, China (Sinovac LS) in comparison with the licensed Olymvax tetanus vaccine. Herein, we report the results of this trial, which provide evidence to support the regulatory submission of Sinovac tetanus vaccine and its potential role in prevention of non-NT diseases.

## 2. Materials and Methods

### 2.1. Study Design and Participants

This randomized, double-blind, positive-controlled Phase I/III clinical trial (ClinicalTrials.gov Identifier: NCT06049940) was conducted to evaluate the safety and immunogenicity of a single dose of Sinovac tetanus vaccine in participants aged 18–44 years in Liangyuan District, Henan Province, between September 2023 and March 2024. The trial was approved by the Independent Ethics Committee of the Henan Provincial Center for Disease Control and Prevention (Nos. 2022-YM-007-02 and 2022-YM-007-04-01) and was implemented in accordance with the requirements of the National Medical Products Administration (NMPA) Registration Certificate (No. 2021LP00901), as well as the principles of the Declaration of Helsinki, Good Clinical Practices (GCPs), and relevant ethical standards.

The trial was conducted in two phases. Following a 7-day safety follow-up of the Phase I trial, the Phase III trial proceeded as no safety risks were observed. A total of 60 participants were enrolled in the Phase I trial, with the participants randomized at a 1:1 ratio to receive either the investigational vaccine or the control vaccine. The Phase III trial adopted the same study design as Phase I, enrolling 1200 participants who were assigned to either the investigational group or the control group. Each participant was followed up for approximately 6 months.

The trial enrolled healthy adults aged 18–44 years. Prior to any study procedures, all the enrolled participants signed written informed consent forms. The main exclusion criteria were as follows: abnormal axillary temperature (>37 °C); a history of previous tetanus infection; receipt of tetanus vaccines or TTCVs within the past 10 years; administration of tetanus immunoglobulin or tetanus antitoxin within the past 6 months; a positive urine pregnancy result; planning to become pregnant within 3 months; or breastfeeding at the time of enrollment.

### 2.2. Randomization and Blinding

For both Phases I and III clinical trials, the participants were randomly allocated at a 1:1 ratio. Each participant was given a unique study code sequentially per their enrollment order, and then administered the vaccine matching that code.

The investigational and control vaccines were indistinguishable regarding the outer packaging, labeling, and volume in order to maintain the blinding of the study. Given the differences in their inner packaging, the personnel responsible for vaccine administration and retrieval signed confidentiality agreements and were barred from engaging in any participant-related activities. All the participants, investigators, and study center personnel remained unaware of both the participants’ study group allocations and vaccine codes correspondences.

### 2.3. Procedure

The investigational vaccine, manufactured by Sinovac LS, was supplied in prefilled syringes, each containing tetanus toxoid at a concentration of no less than 40 IU/0.5 mL. This vaccine was verified by the National Institutes for Food and Drug Control (NIFDC) and issued a certificate of compliance. The control vaccine, also an adsorbed tetanus vaccine, was manufactured by Chengdu Olymvax Biopharmaceuticals Inc., China and supplied in vials, each containing 0.5 mL with a tetanus toxoid concentration of no less than 40 IU/0.5 mL. Both vaccines appeared as uniformly opalescent suspensions. All the vaccines were stored and transported at 2–8 °C, shielded from direct light, with freezing strictly forbidden. Both the investigational and control vaccines were administered via an intramuscular injection into the deltoid muscle of the upper arm, adhering to a single-dose immunization schedule. Vaccinations were performed by trained professionals in accordance with the Standard Operating Procedure (SOP) established at the trial site.

Immediate adverse events (AEs) were observed for 30 min following vaccination. AEs were recorded by the participants using diary cards and contact cards. Solicited local and systemic AEs were documented within 7 days post-vaccination. Unsolicited AEs and serious adverse events (SAEs) were recorded within 30 days and 6 months after vaccination, respectively. The solicited AEs included vaccination site symptoms (e.g., pain, induration, swelling, erythema, rash, pruritus, and cellulitis) and systemic symptoms [fever (axillary temperature), acute allergic reaction, diarrhea, anorexia, vomiting, nausea, myalgia (not at the vaccination site), headache, cough, fatigue, and arthralgia]. The severity grading of AEs was conducted by the investigators in accordance with *The Guidelines for Adverse Event Classification Standards for Clinical Trials of Preventive Vaccines (2019)* issued by the NMPA [12]. The correlation between the AEs and vaccination was determined by the investigators.

Venous blood samples (approximately 3 mL) were collected from the participants in Phase III before and 30 days after vaccination. The tetanus antibody concentration was quantified using an in-house enzyme-linked immunosorbent assay (ELISA) developed and performed by NIFDC. SoftMax Pro software (Version 5.0.1) was used to generate a standard curve via the four-parameter method (with R^2^ > 0.98 required for a valid assay), which was then applied to calculate the tetanus antibody concentration in each serum sample. If the optical density (OD) value of a serum sample fell outside the standard curve range, the sample’s dilution factor was adjusted for retesting. The accuracy and reliability of the detection system were ensured through method validation and quality control based on the detection results of internal reference sera included in each microplate.

### 2.4. Endpoints

The primary endpoints of the Phase I clinical trial were the incidences of adverse reactions (ARs) within 7 days and 30 days post-vaccination. Additionally, the incidence of graded 3 or higher ARs within 30 days post-vaccination was evaluated. The secondary endpoint was the occurrence of SAEs throughout the study.

In the Phase III clinical trial, the primary endpoint was the seroconversion rate of tetanus antibodies at 30 days post-vaccination. The secondary endpoints included the seroprotection rate and long-term seroprotection rate at 30 days post-vaccination, as well as the geometric mean concentration (GMC) and geometric mean increase (GMI) in tetanus antibodies. The secondary endpoints also encompassed all the safety indicators consistent with those evaluated in the Phase I trial. Subgroup analyses were conducted for both the susceptible and non-susceptible subgroups, with the analyses of total and susceptible participants serving as the primary references for the conclusions.

### 2.5. Statistical Analyses

For the Phase I clinical trial, the sample size was determined in line with the relevant technical guidelines for preventive vaccine clinical trials issued by the NMPA [13,14], which suggests 20 to 30 participants as the appropriate sample size for Phase I clinical trials.

The sample size for the Phase III clinical trial was established based on a non-inferiority evaluation, which required that the lower limit of the two-sided 95% confidence interval (CI) for the difference in seroconversion rates (investigational group minus control group) be ≥−5%. The estimated seroconversion rate for tetanus antibodies at day 30 post-vaccination in the control group was at least 99%, which was presumed to be equivalent to that of the investigational group. Using NCSS-PASS software (Version 15.0), the calculated sample size per group was 170 participants, with a two-sided significant level of 0.05, a desired power of 90.0%, and a non-inferiority margin of −5%. Considering that the sample size per group should be no less than 500 [14], with an estimated dropout rate of 20%, the final sample size was 1200 participants (600 per group).

The safety set (SS) included all the participants who received a single dose of vaccine. Immunogenicity assessments were conducted using the per-protocol set (PPS), which comprised all the eligible, randomized participants who adhered to the vaccination schedule and had available antibody results both pre- and post-vaccination. The participants were excluded from the PPS if they met any of the following criteria: (1) protocol deviation, (2) use of vaccines or drugs prohibited by the protocol, or (3) newly diagnosed autoimmune diseases during the study period.

Seroprotection (seropositivity) was defined as an antibody concentration ≥ 0.1 IU/mL [4,15], while long-term seroprotection was defined as an antibody concentration ≥ 1.0 IU/mL [16]. The seroconversion was determined as either a pre-vaccination antibody concentration < 0.1 IU/mL with a post-vaccination concentration ≥ 0.1 IU/mL, or a pre-vaccination concentration ≥ 0.1 IU/mL with a post-vaccination concentration showing at least a fourfold increase. Susceptibility was defined as an antibody concentration < 0.1 IU/mL, and non-susceptibility as an antibody concentration ≥ 0.1 IU/mL [4,15,16].

The AEs were medically coded by MedDRA (Version 26.1), categorized by local and systemic symptoms, and statistically classified by the system organ class (SOC) and preferred term (PT). All AEs were summarized as frequencies and percentages. The incidence rates of AEs were compared using a Chi-square test, corrected Chi-square test, or Fisher’s exact test, as appropriate. The seroconversion rates, seroprotection (seropositivity) rates, and long-term seroprotection rates were calculated with exact 95% confidence intervals (CIs) and compared using a Chi-square test or Fisher’s exact test, as appropriate. The conservative Miettinen & Nurminen method was employed to calculate the difference in seroconversion rates at 30 days post-vaccination, followed by a non-inferiority analysis. For both groups, the GMC of antibodies and the corresponding two-sided 95% CIs were calculated by taking the anti-log of the arithmetic mean and 95% CIs of the log concentration transformations, with the intergroup differences assessed using the log-transformed t-test. The GMIs were calculated as the ratio of the GMC at 30 days post-vaccination to the GMC prior to vaccination. A *p* value less than 0.05 was considered statistically significant for between-groups differences. The statistical analyses were conducted using SAS version 9.4 (SAS Institute Inc., Cary, NC, USA).

## 3. Results

### 3.1. Baseline Demographic Characteristics

**Figure 1:**
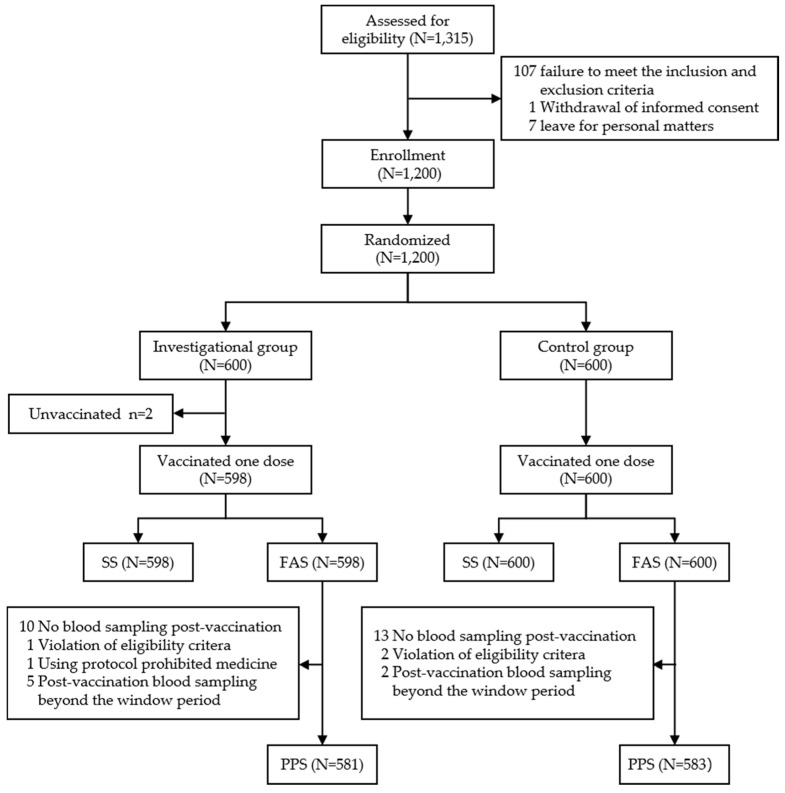
Phase Ⅲ clinical trial profile. All participants received single dose of allocated vaccine according to protocol. SS: safe analysis set; FAS: full analysis set; PPS: per-protocol set. The reasons for no blood sampling post-vaccination were as follows: in the investigational group, 3 withdrawals without any reason and not due to vaccine-related AEs, and 7 due to migration, relocation, or departure from the trial site; in the control group, 2 withdrawals without any reason and not due to vaccine-related AEs; 10 due to migration, relocation, or departure from the trial site; and 1 disconnected.

A total of 66 individuals aged 18–44 years were screened for eligibility, with 60 individuals enrolled in the Phase I clinical trial. All 60 participants completed vaccination and were included in the SS. In the Phase III clinical trial, 1315 individuals were screened and 1200 were recruited, of whom 1164 (581 in the investigational group and 583 in the control group) were included in the PPS. A total of 1198 participants (598 in the investigational group, 600 in the control group) were included in the SS (Figure 1). The baseline demographic characteristics were comparable across both groups (Table 1).

### 3.2. Immunogenicity

In the PPS, the pre-vaccination seroprotection rates were 22.72% in the investigational group and 17.84% in the control group, with long-term seroprotection rates of 0.52% and 0.51%, and GMCs of 0.041 IU/mL and 0.038 IU/mL, respectively; no significant differences were observed between the two groups. At 30 days post-vaccination, the seroconversion rates were 99.48% in the investigational group and 99.66% in the control groups, meeting the non-inferiority criteria with a rate difference of −0.17% (95%CI: −1.20%, 0.78%). The long-term seroprotection rate was higher in the investigational group than in the control group (92.77% vs. 89.88%), with a difference of 2.89% (95% CI: −0.35%, 6.19%), though this difference was not statistically significant (*p* = 0.0798). The investigational group showed a significantly higher GMC compared with that of the control group (4.721 IU/mL vs. 3.627 IU/mL, *p* < 0.0001). At 30 days post-vaccination, the GMIs were 114.66 in the investigational group and 96.56 in the control group.

In the susceptible participants, the pre-vaccination GMCs were the same among the groups (0.026 IU/mL), and the day 30 post-vaccination seroconversion rates were 99.78% and 99.79%, with a non-inferior rate difference of −0.01% (95%CI: −1.06%, 0.97%). The long-term seroprotection rates were 90.65% and 88.10%, with a rate difference of 2.55% (95%CI: −1.46%, 6.55%). The investigational group had a higher GMC compared with that of the control group (3.693 IU/mL vs. 3.205 IU/mL, *p* = 0.0300).

In the non-susceptible participants, the pre-vaccination GMCs were comparable between the groups (*p* = 0.9623). At 30 days post-vaccination, the seroconversion rates were 98.48% and 99.04% in the investigational and control groups, respectively, with a non-inferior rate difference of −0.55% (95%CI: −4.53%, 3.87%). The long-term seroprotection rates were 100.00% and 98.08%, with a rate difference of 1.92% (95%CI: −0.95%, 6.76%). The investigational group had a significantly higher GMC than the control group (10.881 IU/mL vs. 6.412 IU/mL, *p* < 0.0001) (See Table 2).

### 3.3. Safety

In the combined Phase I/III clinical trial, 1258 participants received a single dose of either the investigational vaccine or the positive control. From the start of vaccination to 30 days post-vaccination, the overall incidence of ARs in the investigational and control groups was 12.26% and 13.65%, respectively, with no statistically significant difference (*p* = 0.4631). All the ARs in the investigational group were reported within 7 days post-vaccination. The incidences of local ARs were 9.71% in the investigational group and 12.06% in the control group, showing no statistically significant difference (*p* = 0.1809). The incidences of systemic ARs were also comparable between the groups (3.34% vs. 2.06%, *p* = 0.1614). The severity of ARs were mild (grade 1: 11.31% vs. 13.17%, *p* = 0.3119) or moderate (grade 2: 3.03% vs. 1.27%, *p* = 0.0317), with no grade 3 or higher ARs occurring.

Within 30 days post-vaccination, the most frequently reported local AR was vaccination site pain, followed by swelling, erythema, and pruritus. The most common systematic AR was fever, with an incidence of 1.59% in the investigational group and 0.95% in the control group, showing no significant difference between the groups (see Table 3).

From the initiation of vaccination to 6 months post-vaccination, a total of four SAEs occurred (two in each group); all were assessed as unrelated to vaccination.

## 4. Discussion

This Phase I/III clinical trial met the prespecified non-inferiority endpoints for immunogenicity. At 30 days post-vaccination, the seroconversion rates of antibodies in the total population and susceptible population both demonstrated the non-inferiority of the investigational vaccine compared to the control vaccine, with differences of −0.17% (95%CI: −1.20%, 0.78%) and −0.01% (95%CI: −1.06%, 0.97%), respectively. The GMC of antibodies against tetanus toxoid in the investigational group was higher than that in the control group (4.721 IU/mL vs. 3.627 IU/mL, *p* < 0.0001), suggesting enhanced and potentially more enduring immune protection. The pivotal Phase III clinical trial data for the control vaccine showed a seroconversion rate of 99.83% and a GMC of 3.38 IU/mL [17], which were generally consistent with the results of the present study. These findings indicate that the investigational vaccine was capable of inducing a favorable immune response in both the total population and susceptible population, with antibody levels higher than those induced by the licensed control vaccine.

The pre-immunization seroprotection rates were low in both the investigational (22.72%) and control groups (17.84%), with the long-term seroprotection rates being minimal at 0.52% and 0.51%, respectively. This indicates that the majority of participants without a history of TTCVs vaccination for ten years or longer were tetanus-susceptible. These rates are relatively lower than those reported in a trial conducted in China [17], where participants who had received TTCVs within the previous three years were excluded. This discrepancy may be attributed to the decline in antibody levels with increasing time elapsed since the last dose of TTCVs administered [18]. The majority of participants exhibited tetanus antibody levels below the seroprotective threshold, which indicates unmet immunization needs in this population. These findings not only emphasize the importance of decennial tetanus vaccine booster immunization for adults—a practice recommended in most countries and regions across Europe and North America—but also highlight the value of utilizing tetanus vaccine in adults in the post-traumatic phase, as specified in the *Guideline* [1]. The market launch of Sinovac tetanus vaccine is anticipated to expand the options for post-traumatic tetanus prevention and booster immunization.

The trial demonstrated that the investigational vaccine has a favorable safety profile. The incidence rates of ARs were comparable between the investigational and control groups (12.26% vs. 13.65%, *p* = 0.4631), with the ARs predominantly being mild to moderate. Notably, the ARs in the investigational group occurred within 7 days post-vaccination. Furthermore, fever was the most common systemic AR, with incidence rates of 1.59% in the investigational group and 0.95% in the control group, respectively. This is consistent with reports indicating that approximately 0.5–7% of recipients develop a fever after booster immunization [4]. Although the incidence of grade 2 ARs in the investigational group (3.03%) was slightly higher than that in the control group (1.27%), both incidences were low overall, with the difference remaining below 2%. The factors associated with the incidence and severity of ARs following tetanus vaccination include the pre-immunization antibody levels against the tetanus toxoid, the antigen content, the type and quantity of adjuvants, and the injection route [19,20]. Theoretically, the trial adopted a randomized, double-blind design to control those factors, minimizing their potential influence. However, the pre-immunization seropositivity rate in the investigational group was slightly higher than that in the control group, which might explain the higher incidence of grade 2 ARs in the investigational group. Given the low incidence, this did not present additional safety risks. Moreover, no SAEs related to vaccination were reported. Hence, the safety profile of Sinovac tetanus vaccine is comparable to that of commercially available counterparts, with no unexpected ARs, and risks that are manageable.

This study has several acknowledged limitations. Firstly, the trial only evaluated the immunogenicity of a single dose in adults aged 18–44 years for the non-inferiority assessment of immunogenicity. This context is noteworthy, particularly given that the coverage rate of the third dose of DTP-containing vaccines in China had exceeded 99% by 2021 [21], and the immunoprotective threshold of ≥0.1 IU/mL is widely adopted [4,15,16,22,23]. Against this backdrop, the conventional vaccination schedule recommended by the WHO is less practical for clinical trial design due to the high childhood vaccination rate [4]. Previous studies have demonstrated that TTCVs, including absorbed tetanus vaccines and combination vaccines, can induce robust immune responses and exhibit favorable safety profiles in adults aged 18 years and older [24], with no observed decrease in the immune response with increasing age [25]. These findings support the potential application of the investigational vaccine in populations aged 45 and older. Secondly, this study excluded pregnant women, a high-risk population for tetanus prevention, due to the impracticability of their inclusion in the clinical trial. Post-marketing studies are warranted to evaluate the effectiveness and safety of the investigational vaccine in high-risk populations—specifically including pregnant women and middle-aged/elderly individuals with undocumented or uncertain tetanus vaccination histories—thereby providing additional evidence for the vaccine’s clinical application.

## 5. Conclusions

In conclusion, following administration of a single dose of the tetanus vaccine manufactured by Sinovac LS., adults aged 18–44 years exhibited non-inferior immunogenicity relative to that of the currently marketed control vaccine. The new tetanus vaccine demonstrated a favorable safety profile with no new safety concerns identified. This supports the application of this vaccine for tetanus preventive immunization.

## Figures and Tables

**Table 1:** Baseline demographic characteristics of the participants in Phase Ⅰ (SS) and Phase Ⅲ clinical trial (PPS).

Participants	Characteristics	Investigational Group	Control Group	*p* Value
Phase Ⅰ	No. of participants	30	30	
	Age (years), Mean ± SD	31.26 ± 8.04	33.47 ± 6.75	0.2544
	Male, n (%)	8 (26.67)	12 (40.00)	0.2733
	Han Chinese, n (%)	28 (93.33)	30 (100.00)	0.4720
	Height (cm), Mean ± SD	166 ± 7	166 ± 9	0.9254
	Weight (kg), Mean ± SD	66.1 ± 11.8	72.1 ± 18.4	0.1371
Phase Ⅲ Total Participants	No. of participants	581	583	
	Age (years), Mean ± SD	33.79 ± 6.07	33.31 ± 6.05	0.1782
	Male, n (%)	283 (48.71)	304 (52.14)	0.2412
	Han Chinese, n (%)	561 (96.56)	557 (95.54)	0.3730
	Height (cm), Mean ± SD	166 ± 9	167 ± 9	0.2590
	Weight (kg), Mean ± SD	70.5 ± 14.8	71.5 ± 15.2	0.2391
Phase Ⅲ Non-susceptible participants	No. of participants	132	104	
	Age (years), Mean ± SD	33.01 ± 6.93	33.69 ± 6.28	0.4337
	Male, n (%)	68 (51.52)	59 (56.73)	0.4249
	Han Chinese, n (%)	130 (98.48)	100 (96.15)	0.4759
	Height (cm), Mean ± SD	166 ± 8	168 ± 9	0.1475
	Weight (kg), Mean ± SD	69.7 ± 14.9	73.2 ± 15.7	0.0795
Phase Ⅲ Susceptible participants	No. of participants	449	479	
	Age (years), Mean ± SD	34.02 ± 5.78	33.23 ± 6.00	0.0414
	Male, n (%)	215 (47.88)	245 (51.15)	0.3203
	Han Chinese, n (%)	431 (95.99)	457 (95.41)	0.6616
	Height (cm), Mean ± SD	166 ± 9	167 ± 9	0.5529
	Weight (kg), Mean ± SD	70.7 ± 14.8	71.1 ± 15.5	0.6555

SD: standard deviation. *p* values are calculated using Pearson’s Chi-squared test or Fisher’s exact test.

**Table 2:** Pre- and post-vaccination geometric mean concentrations, seroprotection rates, geometric mean increase, and long-term seroprotection rates of serum tetanus antibodies in total participants, susceptible participants, and non-susceptible participants (PPS).

Participants/Timing	Indicator	Investigational Group	Control Group	Rate Difference (%) or Ratio (95%CI)	*p* Value
**Total Participants**	**Number**	**581**	**583**		
Pre-vaccination	Seroprotection rate, % (95%CI)	22.72 (19.37, 26.35)	17.84 (14.81, 21.19)		0.0384
	Long-term seroprotection rate, % (95%CI)	0.52 (0.11, 1.50)	0.51 (0.11, 1.50)		>0.9999
	GMC (IU/mL) (95%CI)	0.041 (0.038, 0.045)	0.038 (0.034, 0.041)		0.1600
30 days post-vaccination	Seroconversion rate, % (95%CI)	99.48 (98.50, 99.89)	99.66 (98.77, 99.96)	−0.17 (−1.20, 0.78) *	0.9969
	Seroprotection rate, % (95%CI)	99.83 (99.04, 100.00)	99.83 (99.05, 100.00)	0.00 (−0.81, 0.81)	>0.9999
	Long-term seroprotection rate, % (95%CI)	92.77 (90.35, 94.74)	89.88 (87.14, 92.21)	2.89 (−0.35, 6.19)	0.0798
	GMC (IU/mL) (95%CI)	4.721 (4.322, 5.156)	3.627 (3.349, 3.927)	1.302 (1.156, 1.466)	<0.0001
	GMI (95%CI)	114.66 (104.70, 125.57)	96.56 (88.01, 105.94)	——	——
**Susceptible Participants**	**Number**	**449**	**479**		
Pre-vaccination	GMC (95%CI)	0.026 (0.024, 0.028)	0.026 (0.024, 0.028)		0.9116
30 days post-vaccination	Seroconversion rate, % (95%CI)	99.78 (98.77, 99.99)	99.79 (98.84, 99.99)	−0.01 (−1.06, 0.97) *	>0.9999
	Long-term seroprotection rate, % (95%CI)	90.65 (87.57, 93.18)	88.10 (84.86, 90.86)	2.55 (−1.46, 6.55)	0.2093
	GMC (95%CI)	3.693 (3.362, 4.057)	3.205 (2.936, 3.498)	1.152 (1.014, 1.310)	0.0300
	GMI (95%CI)	141.09 (127.96, 155.56)	121.72 (110.80, 133.71)	——	——
**Non-susceptible Participants**	**Number**	**132**	**104**		
Pre-vaccination	GMC (IU/mL) (95%CI)	0.192 (0.172, 0.215)	0.193 (0.171, 0.218)		0.9623
30 days post-vaccination	Seroconversion rate, % (95%CI)	98.48 (94.63, 99.82)	99.04 (94.76, 99.98)	−0.55 (−4.53, 3.87)	>0.9999
	Long-term seroprotection rate, % (95%CI)	100.00 (97.24, 100.00)	98.08 (93.23, 99.77)	1.92 (−0.95, 6.76)	0.1931
	GMC (IU/mL) (95%CI)	10.881 (9.353, 12.659)	6.412 (5.512, 7.459)	1.697 (1.367, 2.106)	<0.0001
	GMI (95%CI)	56.63 (47.40, 67.65)	33.24 (27.76, 39.79)	——	——

* Achieving non-inferiority. Seroconversion rate: For the susceptible participants, the seroconversion rate is defined as the rate of participants with post-vaccination antibodies against tetanus toxoid ≥ 0.1 IU/mL; for the non-susceptible participants, the seroconversion rate is defined as the rate of participants with at least a fourfold increase in antibodies against tetanus toxoid post-vaccination compared with that pre-vaccination. The seroprotection rate is defined as the rate of participants with antibodies against tetanus toxoid ≥ 0.1 IU/mL. The long-term seroprotection rate is defined as the rate of participants with antibodies against tetanus toxoid ≥ 1.0 IU/mL. GMC: geometric mean concentration; GMI: geometric mean increase; PPS: per-protocol set.

**Table 3:** Adverse reactions within 30 days after vaccination (SS).

Adverse Reactions	Investigational Group (N = 628)		Control Group (N = 630)		*p* Value
	Events	Participants (%)	Events	Participants (%)	
**Total adverse reactions**	**148**	**77 (12.26)**	**120**	**86 (13.65)**	**0.4631**
*General disorders and administration site conditions*	*129*	*72 (11.46)*	*109*	*84 (13.33)*	*0.3147*
Fever	10	10 (1.59)	6	6 (0.95)	0.3111
Fatigue	3	3 (0.48)	5	5 (0.79)	0.7262
Vaccination site erythema	22	22 (3.50)	7	7 (1.11)	0.0047
Vaccination site pruritus	18	18 (2.87)	9	9 (1.43)	0.0785
Vaccination site pain	40	40 (6.37)	62	62 (9.84)	0.0241
Vaccination site induration	1	1 (0.16)	3	3 (0.48)	0.6188
Vaccination site ecchymosis	0	0 (0.00)	1	1 (0.16)	>0.9999
Vaccination site swelling	35	35 (5.57)	16	16 (2.54)	0.0064
*Respiratory, thoracic, and mediastinal disorders*	*6*	*3 (0.48)*	*7*	*4 (0.63)*	*>0.9999*
Nasal congestion	0	0 (0.00)	2	2 (0.32)	0.4996
Cough	2	1 (0.16)	3	3 (0.48)	0.6188
Oropharyngeal pain	2	2 (0.32)	0	0 (0.00)	0.2490
Rhinorrhea	0	0 (0.00)	1	1 (0.16)	>0.9999
Sneezing	0	0 (0.00)	1	1 (0.16)	>0.9999
Pharyngeal erythema	1	1 (0.16)	0	0 (0.00)	0.4992
Pharyngeal edema	1	1 (0.16)	0	0 (0.00)	0.4992
*Musculoskeletal and connective tissue disorders*	*5*	*5 (0.80)*	*1*	*1 (0.16)*	*0.2181*
Arthralgia	1	1 (0.16)	0	0 (0.00)	0.4992
Myalgia	3	3 (0.48)	1	1 (0.16)	0.6143
Limb discomfort	1	1 (0.16)	0	0 (0.00)	0.4992
*Metabolism and nutrition disorders*	*1*	*1 (0.16)*	*0*	*0 (0.00)*	*0.4992*
Decreased appetite	1	1 (0.16)	0	0 (0.00)	0.4992
*Gastrointestinal disorders*	*1*	*1 (0.16)*	*0*	*0 (0.00)*	*0.4992*
Diarrhea	1	1 (0.16)	0	0 (0.00)	0.4992
*Nervous system disorders*	*6*	*5 (0.80)*	*2*	*2 (0.32)*	*0.4459*
Headache	6	5 (0.80)	2	2 (0.32)	0.4459
*Immune system disorders*	*0*	*0 (0.00)*	*1*	*1 (0.16)*	*>0.9999*
Hypersensitivity	0	0 (0.00)	1	1 (0.16)	>0.9999

## Data Availability

Requests to access the datasets should be directed to the corresponding authors.

## Abbreviations

The following abbreviations are used in this manuscript:

AE	adverse event
AR	adverse reaction
CI	confidence interval
CDC	US Centers for Disease Control and Prevention
DT	diphtherial and tetanus combined vaccine, adsorbed
DTaP	diphtheria, tetanus, and acellular pertussis combined vaccine, adsorbed
ECDC	European Center for Disease Prevention and Control
ELISA	enzyme-linked immunosorbent assay
FAS	full analysis set
GMC	geometric mean concentration
GMI	geometric mean increase
NIFDC	National Institutes for Food and Drug Control
NMPA	National Medical Products Administration
OD	optical density
PPS	per-protocol set
PT	preferred term
SAE	serious adverse event
SOC	system organ class
SOP	standard operating procedure
SS	safety set
Td	low-dose diphtheria toxoid
TTCVs	tetanus toxoid-containing vaccines
WHO	World Health Organization
